# Risk of binge eating disorder in patients with metabolic dysfunction-associated steatotic liver disease

**DOI:** 10.1007/s40519-023-01628-2

**Published:** 2023-12-06

**Authors:** Lucia Brodosi, Michele Stecchi, Francesca Marchignoli, Elisabetta Lucia, Lucia Magnani, Valeria Guarneri, Maria Letizia Petroni, Giulio Marchesini, Loris Pironi

**Affiliations:** 1 Department of Medical and Surgical Sciences, University of Bologna, Bologna, Italy; 2 Clinical Nutrition and Metabolism Unit, IRCCS-Azienda Ospedaliero-Universitaria Di Bologna, Bologna, Italy; 3 Alma Mater University, Bologna, Italy

**Keywords:** Binge eating disorder, Metabolic dysfunction-associated steatotic liver disease, Metabolic syndrome, FibroScan, Obesity, Overweight

## Abstract

**Purpose:**

Very few data exist on the association between metabolic dysfunction-associated steatotic liver disease (MASLD) and eating disorders. The study aimed to evaluate the presence of binge eating disorder (BED), in MASLD subjects.

**Methods:**

Demographic, clinical investigation, anthropometric measurements and laboratory were collected in 129 patients with MASLD (34.1% males; age, 53.7 years; BMI, 34.4 kg/m^2^) addressed by general practitioners to a hospital-based unit of metabolic disorders. The risk of binge eating was tested by the binge eating scale (BES); values in the range 17–26 were considered “possible” BED, values > 26 were considered “probable” BED. Hepatic steatosis and fibrosis were tested by surrogate biomarkers and imaging (transient elastography). Calorie intake and lifestyle were self-assessed by questionnaires.

**Results:**

Possible BED was present in 17.8% of cases, probable BED in another 7.6%, and were neither associated with gender, obesity class, diabetes, features of metabolic syndrome, nor with presence and severity of hepatic steatosis and fibrosis. Also steatosis grade by CAP and fibrosis stage by liver stiffness did not correlate with BES. However, an association was present between the daily caloric intake and “possible” BED (odds ratio, 1.14; 95% confidence interval, 1.05–1.24; “probable” BED, 1.21; 1.07–1.37), after adjustment for confounders.

**Conclusion:**

Binge eating, as scored by BES, is present in a significant proportion of MASLD cases screened for metabolic disorders in a specialized center. It may impact behavioral treatment, reducing the chance of weight loss without systematic psychological support.

*Level of Evidence*: Level III, cohort analytic study

## Introduction

Steatotic liver disease (SLD) is the new overarching term chosen to include the different aetiologies of steatosis under a single broad term [1]. In this wide category, the term metabolic dysfunction-associated fatty liver disease (MASLD), replacing non-alcoholic fatty liver disease (NAFLD), categorizes those patients with SLD who consume alcohol within defined limits (below 140 g/wk or 210 g/wk for females and males, respectively) and have at least 1 of 5 cardiometabolic risk factors. MASLD represents an important challenge for metabolic and liver units because of several, intertwined reasons. First, the number of cases in the community is extremely high, continues to increase, and cannot be adequately met by the different healthcare systems. A very recent systematic review and meta-analysis reports an increase in the global worldwide prevalence from 25.3% (1990–2006) to 38% (2016–2019) [2], whereas the prevalence of metabolic dysfunction-associated steatohepatitis (MASH), the progressive form of metabolic liver disease, is well above 5% at histology in most countries [3]. The second critical issue is the lack of approved pharmacologic intervention able to modify the natural course of the disease progressing to cardiovascular or liver outcomes. Under these circumstances, behavioral intervention is the sole treatment approved by clinical practice guidelines to reduce inflammation and progressive fibrosis [4–6]. However, lifestyle modifications are difficult to implement and maintain, and their long-term efficacy remains largely unproven.

Calorie restriction is an important component of lifestyle modifications, particularly in individuals with excess body weight, frequently associated with dysfunctional eating, which may also be triggered by the restriction of food intake. Among formal eating disorders (EDs), binge eating disorder (BED) is defined as a condition characterized by episodes of compulsive eating an objectively large amount of food over a discrete period of time, lack of control, discomfort and emotional distress. BED has been recognized as an official psychiatric diagnosis in DSM-5 [7], sometimes requiring specific psychological treatments and/or pharmacotherapy [8]. By definition, individuals with BED do not usually develop compensatory mechanisms to eliminate excess calories, and obesity is a likely outcome, also increasing the risk of type 2 diabetes (T2D) [9]. Very few data exist on the possible association between BED and the former NAFLD classification. In 95 NAFLD patients screened by the binge eating scale (BES), Zhang et al. reported higher than normal scores in 22 (23.1%) and 6 (6.3%) scored ≥ 27, a value highly diagnostic for BED (“probable” BED) [10]. However, no association was found between possible or probable BED and the components of the metabolic syndrome. Canivet et al. reported a 3.6% prevalence of probable BED in 388 subjects with obesity admitted for bariatric surgery, without any relation with the presence and severity of MASLD [11]. In addition to this, Forlano et al. reported an association between BED, tested by the self-administered BED screener-7 (BEDS-7) questionnaire and sarcopenia in NAFLD, without any relation with liver disease severity, tested by non-invasive markers of fibrosis [12].

The role of BED as a possible driver of the association between calorie intake, liver fat accumulation and NAFLD severity remains scarcely defined. The present study was aimed to determine the prevalence of binge eating risk in patients with metabolic risk factors, as a pre-requisite to plan adequate intervention strategies.

## Materials and methods

### Study design

The present study is part of the ongoing program “One day screening of NASH”, a study started in June 2021 at the IRCCS AOUBO (University of Bologna) aimed at evaluating the presence of hepatic steatosis and the probability of liver fibrosis, measured with non-invasive methods, in a population with suspected metabolic syndrome and referred to our Institution by general practitioners or specialists of other clinical areas without any history, or signs and symptoms of advanced liver disease. According to the program, subjects entering the program are visited by a specialist in metabolic disorders and are screened for SLD by non-invasive methods.

The study was approved by the ethical committee of Area Vasta Emilia Centro (Study 110/2021/Sper/AOUBo); all subjects signed the informed consent to participate in the study and to report publication.

### Methods

All subjects were tested when first seen at our Institution, before any intervention. After anamnesis to exclude secondary causes of steatosis, demographic investigation using a pre-defined set of questions, a clinical examination was carried out to verify the presence of signs of advanced liver disease and anthropometric measurements are carried out according to standardized procedures (weight to half a kilogram, height and waist circumference to half a centimeter). Waist circumference was measured at the midpoint between the last rib and the iliac crest with the subject in an upright position, with feet parallel, the abdomen relaxed and exposed, the arms hanging at the sides of the body. BMI was calculated weight (kg)/height (m)^2^. Based on BMI, the patients were classified as overweight (25–29.9), obesity class I (30–34.9), class II (35–39.9) and class III (≥ 40). During the interview, special care was taken to investigate alcohol consumption in order to correctly classify patients as MASLD vs. Met-ALD, the new category including SLD patients with both at least one cardiometabolic risk factor and alcohol intake per week between 140–350 g/week and 210–420 g/week for females and males, respectively.

The diagnosis of metabolic syndrome was based on the redefinition of the National Cholesterol Education Program—Adult Treatment Panel III (NCEP-ATPIII) was used [13], which requires the presence of at least 3 of the following five criteria: (a) waist circumference ≥ 94 cm in men and ≥ 80 in women; (b) triglycerides ≥ 150 mg/dL or ongoing lipid-lowering therapy; (c) HDL-cholesterol ≤ 40 mg/dL in men and ≤ 50 in women; (d) systolic blood pressure ≥ 130 mmHg and diastolic pressure ≥ 85 or current antihypertensive therapy; (e) fasting blood sugar greater ≥ 100 mg/dL or diagnosis of diabetes mellitus.

Blood samples were drawn with patients fasting for at least 12 h, for the determination of a panel of laboratory parameters, including those needed for the assessment of surrogate biomarkers of steatosis and fibrosis, if not available within 90 days before the visit. Laboratory tests were performed by the Metropolitan Laboratory Service of Bologna, which provides all analyses performed by public services in the area. The upper limits of alanine aminotransferases (ALT) were set at 30 U/L in men and 19 U/L in women [14]. The Fatty Liver Index (FLI) was calculated as marker of steatosis using the formula: FLI = (e^0.953 × log_e_(triglycerides) + 0.139 × BMI + 0.718 × log_e_(GGT) + 0.053 × waist circumference − 15.745) / (1 + e^0.953 × log_e_(triglycerides) + 0.139 × BMI + 0.718 × log_e_(GGT) + 0.053 × waist circumference − 15.745) × 100. Based on the FLI score, patients were divided into three categories: low risk of steatosis (FLI < 30), intermediate risk of steatosis (FLI between 30 and 60) and high risk of steatosis (FLI > 60) [15].

Similarly, the presence of advanced fibrosis was estimated using the Fibrosis-4 index (Fib-4), using the formula described in the literature: Fib-4 = (age × AST)/platelet count (× 10^9^/L) × ALT) [16]. Fib-4 values < 1.30 were considered to exclude advanced fibrosis, values > 2.67 were defined as high risk of advanced fibrosis, in the range between 1.30 and 2.67 were considered indeterminate [17].

Finally, all patients were tested by vibration-controlled transient elastography (VCTE) using FibroScan™ (Echosense, Paris, France). Both liver stiffness (marker of fibrosis) and the Controlled Attenuation Parameter (CAP), marker of steatosis, were determined [18]. M or XL probes were used according to what is indicated by the machine itself. The probes undergo calibration every six months. The test was considered reliable if ten valid measurements were achieved with a success rate above 60% and the ratio between the interquartile range and the median (IQR/M) was < 0.3. The stiffness values used were 7.0 kPa, 8.7 kPa and 10.3 kPa to indicate significant fibrosis (F2), advanced fibrosis (F3) and cirrhosis (F4), respectively. The CAP score diagnostic cut-offs were set at 248 dB/m, 260 dB/m and 280 dB/m, respectively, for grade 1 (S1), grade 2 (S2) and grade 3 (S3) steatosis.

### Questionnaires

*Binge Eating Scale (BES).* BES is a 16-item self-administered questionnaire measuring the risk and severity of binge eating [19]. BES examines both behavioral manifestations (eating large amounts of food) and feeling/cognition during possible binge episodes (loss of control, guilt, fear of being unable to stop eating). For each question, the response is graded from 0 to 3; formal BED is considered “unlikely” if the total score sums ≤ 16, “possible” (score between 17 and 26), and “probable” (score ≥ 27). The Italian version of BES was validated by the NetWorking Team Group of the Italian Society for Eating Behavior Disorders (SIS-DCA).

*“Quanto Mangio Veramente”* (How much do I really eat; QMV, 20 items). The questionnaire semi-quantitatively estimates calorie intake on the basis of the habitual weekly consumption and portion size (on a 5-point Likert scale) of 18 items referred to habitual food intake, and a final item on the number of meals not consumed at home during the week [20]. To help subjects with the determination of portion size, pictures are presented to visually explain what is considered small-sized, medium-sized, or large-sized, whereas a few questions specifically investigate the number of specific items consumed during the week, if any (e.g., number of sugar cubes or sugar coffee-spoons, candies, chocolate tablets, alcoholic beverages). Each portion size is given an estimate (value) of its energy content (as multiple of 50 kcal, to simplify calculations). The in-house developed questionnaire has been validated vs. dietary interview carried out by an expert dietitian [21] and has been extensively used since 2006 by specialists and by general physicians in the area of Bologna [22]. All questionnaires had written instructions for self-assessment and were rigidly self-compiled before the visit to limit the influence of healthcare personnel.

### Statistical analysis

The vast majority of tested variables were not normally distributed in the population (Shapiro–Wilk test). Accordingly, data are presented as median (interquartile range—IQR).and percentage for categorical variables. Comparison between different groups was carried out by Student’s t test for unpaired data or Chi-square values, as appropriate. Non-parametric analyses (Mann–Whitney or Wilcoxon test) were also performed. Correlation analysis and/or linear and logistic regression analysis were used to test the association between BES scores or risk of BED and clinical data, with/without adjustment for confounders.

Throughout the text, data are expressed as mean ± SD or percentage, unless otherwise mentioned.

## Results

### Demographic and clinical data

In the period between June 2021 and May 2023, 141 consecutive patients were enrolled, and 129 fit the criteria for SLD based on FLI and/or VCTE values, whereas in 12 both FLI and VCTE did not support the presence of steatosis. Their demographic, clinical, laboratory and VCTE data, as well as the habitual calorie intake of the SLE cohort, are reported in Table 1. Forty-four were male, 85 were women, in the age range between 20 and 81 years. No differences in civil status, smoking habits and education were observed. During clinical investigation, 40% of cases rated their habitual food intake as normal, whereas only 14% of men and 17% of women considered their dietary habits more or less restrictive.

**Table 1:** .

	All cases (*n* = 129)
Demography	
Age (years)	56 (16)
Civil status (single/married/ widowed) (%)	33.6/60.9/5.5
Education (Primary/secondary/ vocational/ degree) (%)	1.8/21.8/47.3/29.1
Self-assessed lifestyle habits	
Food intake (1 to 5) (%)°	1.8/14.7/40.4/35.8/7.3
Physical activity (1 to 5) (%)^	23.8/40.4/23.9/11.9/-
Smoking (yes/no/ex) (%)	27.8/47.2/25.0
Clinical data	
BMI (kg/m^2^)	33.1 (4.1)
Overweight/obesity I, II, III (%)	4.7/64.3/20.9/10.1
BMI at age 20 (kg/m^2^)	23.2 (5.8)
Waist circumference (cm)	105 (14)
Diabetes (%)	17.1
Blood glucose (mg/dL)	94 (17)
Triglycerides (mg/dL)	136 (93)
HDL-cholesterol (mg/dL)	53 (15)
Hypertension (%)	52.7
Systolic blood pressure (mmHg)	125 (25)
Diastolic blood pressure (mm/Hg)	80 (10)
Metabolic syndrome (%)	54.8
No. of features (1, 2, 3, 4, 5) (%)	17.6/27.2/31.2/21.6/2.4
Alanine aminotransferases (U/L)	27 (20)
Normal ALT (%)^#^	33.3
Aspartate aminotransferases (U/L)	24 (13)
γ-Glutamyl transpeptidase (U/L)	33 (38)
Biomarkers and imaging	
Fatty Liver Index (%)	86 (19)
< 30/31–60/ > 60 (%)	– /5.4/94.6
Fibrosis-4 score	0.92 (0.68)
< 1.30/1.31–2.67/ > 2.67 (%)	69.7/27.0/3.3
Controlled attenuation parameter (dB/m)	286 (75)
S0/S1/S2/S3 (%)	18.6/10.9/17.1/53.5
Liver stiffness	4.8 (2.2)
F0-F1/F2/F3/F4 (%)	81.4/7.8/6.2/4.7

*Data are presented as median (interquartile range) or as percent, as appropriate

°Self-graded from 1 to 5 as: very low, low, normal, higher-then-normal, much higher-than-normal

^Self-graded from 1 to 5 as: very sedentary, sedentary, normal, moderately active, very active

^#^ALT, alanine aminotransferase: normal values, < 31 U/L in men, 20 U/L in women

Regarding physical activity, 66% of cases reported a sedentary lifestyle (very sedentary in 27% of women); the proportion of active patients was low in both groups, and none considered themselves very active. All individuals were in the overweight/obesity range, with most of them (64.3%) in obesity class I; in most cases, obesity had developed in the course of life after late adolescence, and BMI at age 20 was referred as 10 kg/m^2^ lower than present BMI. Diabetes had been diagnosed in 17.1% of patients; 54.8% met the criteria for the diagnosis of metabolic syndrome, but a higher percentage of women were free of metabolic features. As expected, waist circumference was smaller (*P* < 0.001), and HDL-cholesterol was significantly higher (*P* < 0.010) in women. Hypertension was more prevalent in men and systolic blood pressure was nearly 10 mmHg higher (*P* < 0.001).

As to liver disease biomarkers, no sex differences in liver enzymes were observed, but a higher number of men had aminotransferase levels within the normal range when graded according to the updated limits [14]. FLI fulfilled the criteria of steatosis in over 92% of the total sample (100% and 88% of men and women, respectively; *P* = 0.018), but all the remaining cases were in the indeterminate range; CAP was indicative of steatosis of a variable degree of severity in over 80% of cases, without gender differences. FIB-4 score was significantly higher in men [median, 1.16 (IQR, 0.81) vs. 0.87 (0.55) in women; *P* = 0.019, Mann–Whitney test], where values were indicative of advanced fibrosis in nearly 10% of cases. On the contrary, in women FIB-4 ruled out advanced fibrosis in nearly 80% of cases and only 20% were in the intermediate class (P between gender, < 0.001). These differences were partly confirmed by transient elastography, with many more women diagnosed without significant fibrosis (F0-1, 95.3% vs. 77.4% in men; *P* = 0.012). Notably, 2 cases in women and 4 cases in men (all confirmed by high FIB-4 values) were diagnosed as fibrosis stage F4, without clinical signs of cirrhosis.

### Food intake

The habitual food intake, measured by the questionnaire, totalled 1990 ± 605 kcal/day in the whole cohort and moderately higher in men vs women (2143 ± 548 kcal/day vs 1911 ± 621; *P* = 0.038). The estimated calorie intake was strictly correlated with the self-assessed evaluation of eating pattern (on a scale from “much lower than normal” to “much higher than normal”) (r = 0.454; *P* < 0.001). On the other hand, there was no correlation between the estimated food intake and the reported physical activity/sedentariness assessment. No correlation was found between calorie intake and the different obesity grades, the presence of MetSyn or T2D and/or the presence and severity of steatosis, as measured by CAP.

### Binge eating

**Fig. 1:**
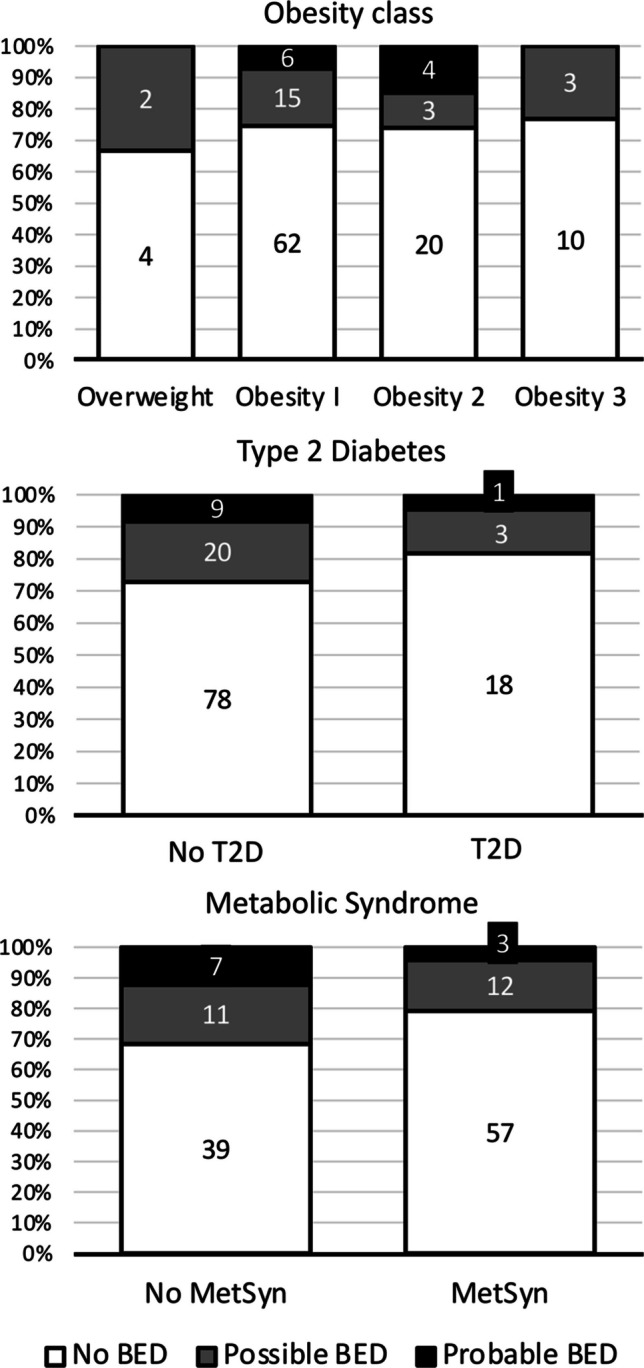
Distribution of cases with suspected eating disorder (possible BED) and with BES score diagnostic for BED (probable BED) in the NAFLD cohort, in relation to the presence of metabolic disorders

**Fig. 2:**
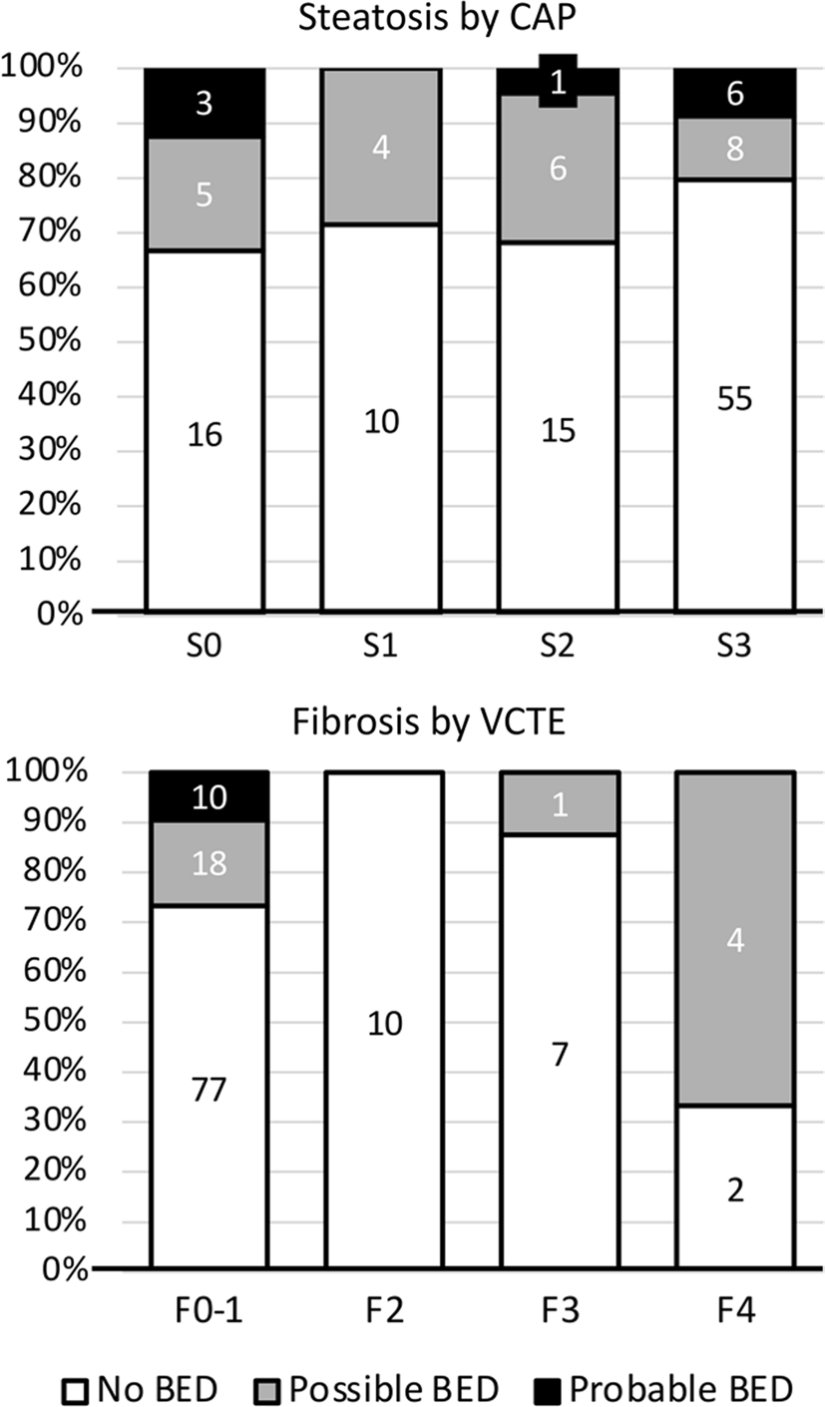
Distribution of cases with suspected eating disorder (possible BED) and with BES score diagnostic for BED (probable BED) in the NAFLD cohort, in relation to the grade of steatosis and the stage of fibrosis. Steatosis grade and fibrosis stage were both measured at FibroScan™ by the controlled attenuation parameter (CAP) and by vibration-controlled transient elastography (VCTE)

**Fig. 3:**
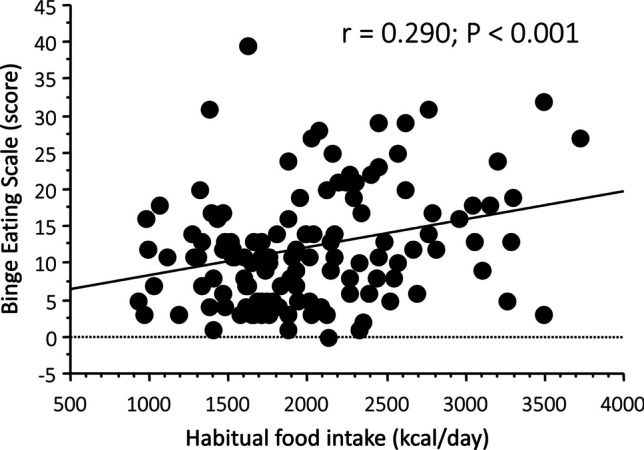
Correlation between BES score and habitual calorie intake, measured by questionnaire, in the NAFLD cohort

BES scores covered a wide range of values (from 0 to 40; median 11, IQR 11), and were not different between men (10, IQR 9) and women (11, IQR 11) (*P* = 0.165, Mann–Whitney test). Scores suspected for binge eating (“possible” BED: range 17–16) were registered in 23 individuals (17.8%, 17 women and 6 men), and in 10 cases more (7.8%, 8 women, 2 men) they exceeded the cut-off of 26 (“probable” BED). BES scores were neither correlated with sex (*P* = 0.343), nor with the principal metabolic abnormalities (obesity class, presence of T2D, presence of MetSyn—Fig. 1). Also the grade of steatosis by CAP (*P* = 0.329) and the stage of fibrosis by transient elastography (*P* = 0.222) failed to correlate with BES class (Fig. 2). Cases of probable BED were randomly distributed across steatosis grade, whereas all cases of probable BED were included among the fibrosis stage F0-1. However, a correlation was present between the habitual food intake and BES class (Kruskal–Wallis test) or BES score (r = 0.290; *P* < 0.001 for both) (Fig. 3). Of note, the BES class correlated with the self-assessed estimate of eating habits (*P* = 0.008) but not with the self-assessed estimate of physical activity/sedentariness (*P* = 0.334).

By logistic regression analysis, the risk of both “possible” BED [odds ratio (OR), 1.12 per 100 kcal of food intake; 95% confidence interval (CI), 1.04–1.21; *P* = 0.004] and of “probable” BED (OR, 1.14; 95%CI, 1.03–1.27; *P* = 0.014) were significantly associated with habitual calorie intake. In multivariable regression, data did not remarkably change after adjustment for multiple confounders (age, sex, BMI, presence of diabetes and MetSyn, severity of steatosis and fibrosis by CAP and transient elastography) (OR for “possible” BED, 1.14; 95% CI, 1.05–1.24; OR for “probable” BED, 1.21; 95% CI, 1.07–1.37).

In a sensitivity analysis performed in the 95 MASLD cases identified by CAP scores ≥ 248 db/m with alcohol intake within the defined limits in the presence of at least 1 of 5 cardiometabolic risk factors, no systematic differences were observed, compared with the whole dataset. The number of cases identified as No-BED/Possible/Probable BED were, respectively, for each category 80, 18 and 7 (76.2%, 17.1% and 6.7%), again with no association with the severity of steatosis or fibrosis (not reported in detail).

In addition to this, no significant associations were observed when the risks of possible or probable BED were tested in three separate models, including demographic variables (age and sex) or the presence of metabolic disorders (BMI/obesity class, MetSyn, DM2) or the severity of liver disease (CAP and FibroScan™ or FLI and FIB-4, either as continuous or as categorical variables).

## Discussion

The study identifies a risk of BED in a proportion of patients with suspected MetSyn, screened for the presence of MAFLD. The proportion of cases with BES scores in the upper range diagnostic for BED, however limited, may constitute a significant additional problem when NAFLD individuals are referred to clinicians for behavior therapy. The estimated prevalence (17.8%, “possible”; 7.8%, “probable”) is definitely higher than what is expected for the general Italian population, which was estimated using a structured interview to be a mere 0.5% [23]. No systematic data are available for BES scoring in the adult population. However, concerning the population affected by obesity, the data reported for “possible BED” were 26.3%, and 13.0% came out as “probable BED” [24].

A definite diagnosis of BED should not be confidently based on the sole score of BES. BES questions cover eating behavior during the previous 3 months and do not consider the number of binging episodes. A correct diagnostic procedure would need a semi-structured interview (Eating Disorder Examination (EDE))[25] or its self-administered version (EDE questionnaire, EDE-Q)[26], which is limited in our setting to patients requiring intervention with the suspect of eating disorder, not during admission for metabolic diseases. However, since the number of episodes for the diagnosis of BED was reduced in DSM-V from two to only one, although in a more restricted (28 days) time frame, it is very likely that also several cases scored high in the “possible” BED category might ultimately be classified as true BED cases; this would increase the need of psychological support for behavior treatment of NAFLD to nearly one-third of cases with overweight/obesity. A debate also exists about the possibility to disentangle BED from food addiction, both characterized by the loss of control on food [27].

According to the NICE Guidelines for eating disorders, the presence of BED would indeed require a therapeutic approach totally different from that of obesity [28]. BED patients should be advised that any attempt to lose weight via dietary restriction, the accepted treatment of MAFLD [4–6], conflicts with the presence of binging episodes; weight loss is not the target of a BED-focused guided self-help program or group-based cognitive-behavioral therapy for eating disorders (CBT-ED), the accepted interventions for this disordered eating pattern. This issue is even more challenging when dealing with MASLD treatment; most patients explicitly ask for drug treatment for their liver disease or, at least, a specific diet able to favor weight loss. At best, dietary intervention should aim to improve adherence to the Mediterranean diet, able to reduce steatosis also in the absence of weight loss [30]. Any attempt to replace weight loss with counselling towards improved lifestyle may be perceived as inadequate to their needs, leading to attrition. A recent systematic review of weight management in BED patients concluded that people with binge eating behaviors confront multiple barriers regarding weight loss, weight loss maintenance and attrition [31]. In a few cases, binge behaviors may also emerge in the course of weight loss programs [32]. This makes psychological support mandatory during the intervention, considering that attrition is reported as a critical issue during the behavior treatment of MASLD [29].

We could not find any association between BES score or category and the presence of metabolic disorder or the severity of liver dysfunction. By definition, BED is not accompanied by purging episodes and the accumulation of calories during binging episodes is the reason for the association of BED with obesity grades, as also confirmed in an Italian population with obesity [33] and in patients with T2D [34]. In the present population, free living and with two-thirds of cases with obesity grade I, we failed to confirm this association, but BES score and category remained strictly linked with habitual dietary intake. Notably, this population was apparently well aware of problematic eating pattern, as demonstrated by the comparison between personal calorie intake and the habitual food intake of other mates or friends.

This awareness is of particular interest, considering the stigma associated with obesity and binge episodes, that promote social isolation and reinforce obesity [35]. For this reason, group-based CBT is particularly suggested by guidelines [28], but this type of treatment may be hardly accepted by free-living persons due to their job involvements and may be difficult to arrange by services outside working hours. This constitutes an even harder challenge for hepatology services, where psychologists are rarely part of the team. We need to strengthen the relation between the different nods involved in MASLD care (hepatology, obesity, diabetes Units) to favor appropriate treatment [4, 36, 37]. In Italy, the three principal associations of hepatology, diabetes and obesity issued a common guideline to promote cooperation [38], and in various centers shared protocols have been developed.

Unfortunately, the awareness of the risk associated with silent liver disease in MASLD remains low, and also in the present series, six patients were seen with surrogate markers compatible with fibrosis F4 (also confirmed by transient elastography) when first screened in a specialized center.

This study has both strengths and limitations. The main strength is the selection of consecutive cases, not specifically addressed to our institution because of a previously diagnosed SLD—which might be subject to selection bias—but addressed for general diagnostic assessment in the presence of altered metabolic parameters. Limitations include the use of surrogate biomarkers such as FLI and Fib-4, a questionnaire to assess BED and a self-assessed questionnaire on lifestyle (subject to information bias). In addition to this, the assessment of alcohol intake was not confirmed by validated measures, but it was tested for comparison with the dietary intake questionnaire, which also comprises the weekly amount of alcohol.

The newly proposed nomenclature of MASLD [1] widened the importance of metabolic factors in steatosis and possibly in the silent progression to cirrhosis, also in the presence of normal aminotransferase levels, as was in the present setting. Only a larger involvement of general practitioners and their proactive, early intervention and referral might help intercept these cases and provide optimal treatment to a cohort at risk of hepatic complications and mortality.

### What is already known on this subject?

Metabolic dysfunction-associated steatotic liver disease (MASLD) is a pathology currently treated with weight loss, achieved by reducing caloric intake and increasing physical activity.

### What does this study add?

In patients suffering from MASLD, the risk of binge eating disorder estimated through the binge eating scale questionnaire appears to be significantly greater than in the general population. Given that a restrictive diet could worsen the eating disorder, before prescribing it for the treatment of MASLD, it is advisable to investigate the presence of BED and, if present, direct the patient to cognitive behavioral therapy at a center specialized in the treatment of eating disorders.

## Data Availability

The datasets generated during and/or analyzed during the current study are available from the corresponding author on reasonable request.
